# Hyperglycemia in Pregnancy-Associated Oxidative Stress Augments Altered Placental Glucose Transporter 1 Trafficking via AMPKα/p38MAPK Signaling Cascade

**DOI:** 10.3390/ijms23158572

**Published:** 2022-08-02

**Authors:** Shuxian Wang, Jie Ning, Jing Huai, Huixia Yang

**Affiliations:** 1 Department of Obstetrics and Gynaecology, Peking University First Hospital, Beijing 100034, China; wsx979@bjmu.edu.cn (S.W.); ningjie1996@pku.edu.cn (J.N.); huaijing@bjmu.edu.cn (J.H.); 2 Beijing Key Laboratory of Maternal Fetal Medicine of Gestational Diabetes Mellitus, Beijing 100034, China

**Keywords:** hyperglycemia in pregnancy, oxidative stress, glucose transporter 1, AMPKα, p38MAPK

## Abstract

GLUT1, being a ubiquitous transporter isoform, is considered primarily responsible for glucose uptake during glycolysis. However, there is still uncertainty about the regulatory mechanisms of GLUT1 in hyperglycemia in pregnancy (HIP, PGDM, and GDM) accompanied by abnormal oxidative stress responses. In the present study, it was observed that the glycolysis was enhanced in GDM and PGDM pregnancies. In line with this, the antioxidant system was disturbed and GLUT1 expression was increased due to diabetes impairment in both placental tissues and in vitro BeWo cells. GLUT1 responded to high glucose stimulation through p38MAPK in an AMPKα-dependent manner. Both the medical-mediated and genetic depletion of p38MAPK in BeWo cells could suppress GLUT1 expression and OS-induced proapoptotic effects. Furthermore, blocking AMPKα with an inhibitor or siRNA strategy promoted p38MAPK, GLUT1, and proapoptotic molecules expression and vice versa. In general, a new GLUT1 regulation pathway was identified, which could exert effects on placental transport function through the AMPKα-p38MAPK pathway. AMPKα may be a therapeutic target in HIP for alleviating diabetes insults.

## 1. Introduction

Oxidative stress (OS) is defined as a disturbance in the equilibrium status of pro-oxidants and antioxidants. A hyperglycemic environment such as pre-gestational diabetes (PGDM) and gestational diabetes mellitus (GDM) could initiate OS, which is reflected by the overproduction of reactive oxygen species (ROS) and defects in the antioxidant defenses [1,2,3]. During normal pregnancies, specific adaptations of maternal nutrient metabolism such as carbohydrates are required to meet the increasing energy needs of both mother and fetus. These variations are superimposed in hyperglycemia in pregnancy (HIP) and subsequently affect placenta transport functions and fetal programming for disease in adulthood [4,5]. Glucose is the principal energy substrate for fetal development and is transported from maternal circulation due to the low production capacity in the fetus [6]. The glucose uptake is dominantly mediated by facilitative transporter proteins in trophoblast cells. 

Glucose transporter 1 (GLUT1) is ubiquitously expressed and is the major glucose transporter in the human placenta [7]. It plays a key role in the primary utility of glucose, namely glycolysis, to generate energy. Altered GLUT1 expression is discovered in pathological pregnancies, implicating abnormal glucose usage. Available documents suggest that GLUT1 was increased in HIP with or without a large infant [8,9,10,11,12,13]. In contrast, its expression is shown to be decreased in IUGR [14]. Given that ROS production or elimination is strongly associated with glycolysis and the subsequent metabolic pathways, the high glucose exposure is often accompanied by ROS accumulation and thus recapitulates the plausible relationships between hyperglycemia and increased metabolic activity through cellular stress-related mechanisms [15]. However, the GLUT1 regulation patterns with exaggerated OS in HIP were unclear.

AMP-activated protein kinase (AMPK), a serine/threonine kinase, regulates the cellular and whole-body energy metabolism under stress conditions, which is inactivated in GDM or T2DM due to the enriched cellular ATP [16]. AMPK is necessary for nutrient transportation and GLUT regulation [17]. Recent studies demonstrated that it could stimulate glucose uptake through GLUT3 in the placenta [18] and GLUT4 in the skeleton muscle independent of insulin [19]. The ability is heightened by treating with the AMPKα agonist [20,21]. Moreover, in the placenta of GDM with macrosomia, AMPKα phosphorylation and the GLUT1 expression level are more decreased and upregulated, respectively, than in GDM with normal birth weights or normal pregnancies [22]. Apart from the traditional view as a sensor of energetic status, AMPKα may be equally important in the regulation of cell proliferation lying downstream of LKB1 [23]. However, the precise mechanism by which AMPK regulates GLUT and cell growth or apoptosis remains unknown.

OS has been involved in regulating molecular pathways in many diseases through p38 mitogen-activated protein kinase (p38MAPK), a stress-activated protein serine/threonine kinase [24]. Recently, researchers suggested that p38MAPK could mediate glucose uptake and exerts beneficial effects appearing to be AMPKα-dependent [25]. AMPKα displays a close relationship with p38MAPK in high glucose-related apoptosis [26], glucolipid metabolism [27], tumor cell survival, and metastasis [28]. Considering the biological similarities between the placenta and malignant tumors such as the microenvironment heterogeneity, a high proliferative rate, and aerobic glycolysis [29], it was hypothesized that GLUT1 is regulated through AMPKα-p38MAPK signaling and may exert influences on placental transport function. 

HIP (GDM and PGDM) constitutes one of the most common metabolic disorders in obstetric populations. PGDM insulting at the beginning of gestation could exert long-term effects on placental development, and GDM foremost leads to functional changes insulting at a later stage of gestation [30]. Therefore, the aim of this study was to investigate the influences of different high-glucose intrauterine environments on placenta transport functions and the regulatory mechanisms of GLUT1 in the context of HIP-induced OS enhancement. It was found that, in HIP groups, the antioxidant substances were decreased concomitantly with overexpressed proapoptotic molecules. GLUT1 expression was also increased and could be regulated by AMPKα-p38MAPK cascades.

## 2. Results

### 2.1. Participant Characteristics

The clinical characteristics of all subjects are summarized in Table 1. A total of 43 pregnancies were enrolled in this study, including 14 normal pregnancies (Control), 10 diet-controlled GDM (GDM1), 9 insulin-controlled GDM (GDM2), and 10 PGDM (Type 2 diabetes). Women with HIP, especially GDM2 and PGDM, exhibited significantly high p-BMI (Control: 21.68 ± 1.765 vs. GDM1: 22.27 ± 2.491 vs. GDM2: 26.56 ± 3.092 vs. PGDM: 25.77 ± 4.803 kg/m^2^, *p* < 0.01), third-trimester fasting glucose level (Control: 4.48 ± 0.371 vs. GDM1: 4.71 ± 0.463 vs. GDM2: 5.17 ± 0.925 vs. PGDM: 5.19 ± 0.838, *p* < 0.05), and decreased gestational weight gain (GWG, Control: 13.37 ± 4.239 vs. GDM1: 11.40 ± 2.989 vs. GDM2: 9.49 ± 2.875 vs. PGDM: 8.45 ± 3.218 kg, *p* < 0.01) when compared with the control group. Moreover, there were significant differences in the OGTT results among the control, GDM1, and GDM2 pregnancies (GLU0: 4.51 ± 0.180 vs. 5.17 ± 0.415 vs. 5.53 ± 0.420 mmol/L, *p* < 0.0001; GLU1: 7.88 ± 1.161 vs. 10.23 ± 1.335 vs. 10.00 ± 1.321 mmol/L, *p* < 0.0001; GLU2: 6.57 ± 0.873 vs. 9.21 ± 1.086 vs. 8.15 ± 1.520 mmol/L, *p* < 0.0001; AUC: 13.42 ± 1.495 vs. 17.42 ± 1.402 vs. 16.84 ± 1.952 mmol/L, *p* < 0.0001). The PGDM pregnancies were under poor control from the first trimester, and the glycosylated hemoglobin (%) and glycated albumin (%) levels were 6.56 ± 1.19/16.71 ± 2.34, 5.85 ± 0.34/16.26 ± 1.78, and 5.82 ± 0.56/16.19 ± 3.21 in the first, second, and third trimesters, respectively. Other clinical factors were similar and of no significant differences.

### 2.2. The Antioxidant Capacity Was Compromised in GDM and PGDM Pregnancies

**Figure 1:**
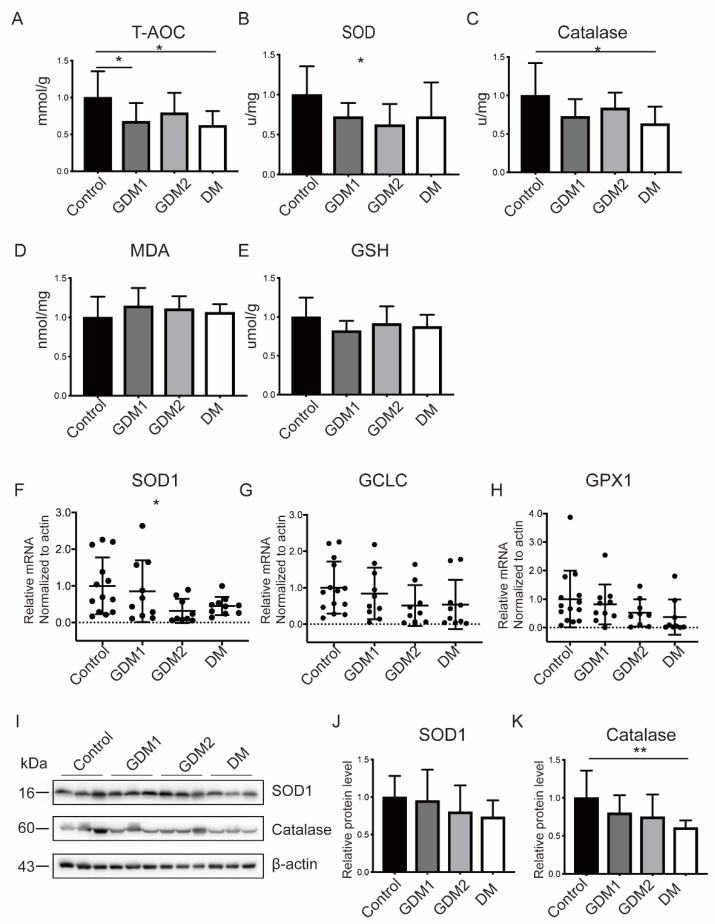
The balance between oxidant and antioxidant substances was disturbed in HIP pregnancies. (**A**–**E**) Changes in the capacity and activity of oxidative stress-related molecules. (**F**–**K**) Placental mRNA and protein levels of antioxidant molecules. Actin served as the internal controls. Data are the mean ± SEM. * *p* < 0.05 and ** *p* < 0.01 by a one-way ANOVA test, followed by a post hoc test.

To explore the effects of hyperglycemia on OS responses, the relevant markers in placenta tissues were detected. It could be observed that the T-AOC capacity (Control: 0.26 ± 0.09 vs. GDM1: 0.17 ± 0.06 vs. GDM2: 0.20 ± 0.07 vs. PGDM: 0.16 ± 0.05 mmol/g, *p* < 0.05), activity assays of SOD1 (Control: 7.49 ± 2.65 vs. GDM1: 5.40 ± 1.30 vs. GDM2: 4.66 ± 1.95 vs. PGDM: 5.41 ± 3.23 U/mg, *p* < 0.05), and catalase (Control: 5.02 ± 2.12 vs. GDM1: 3.64 ± 1.14 vs. GDM2: 4.19 ± 1.02 vs. PGDM: 3.17 ± 1.12 U/mg, *p* < 0.05) were obviously decreased in HIP pregnancies and deteriorated in PGDM (Figure 1A–C). Increased MDA content was present in HIP when compared with the control group but without significant differences (Figure 1D). Consistently, the mRNA (Figure 1F–H) and protein (Figure 1I–K) expression profiles of the antioxidative stress molecules were increased in the normal pregnancies, followed by GDM1, GDM2, and PGDM pregnancies. Moreover, it could be observed that OS-induced apoptosis was increased in the HIP groups and most evident in the GDM2 or PGDM group (Supplementary Figure S1A–D). 

### 2.3. Glucose Metabolism Was Disrupted in Placentas of Women with Hyperglycemia

**Figure 2:**
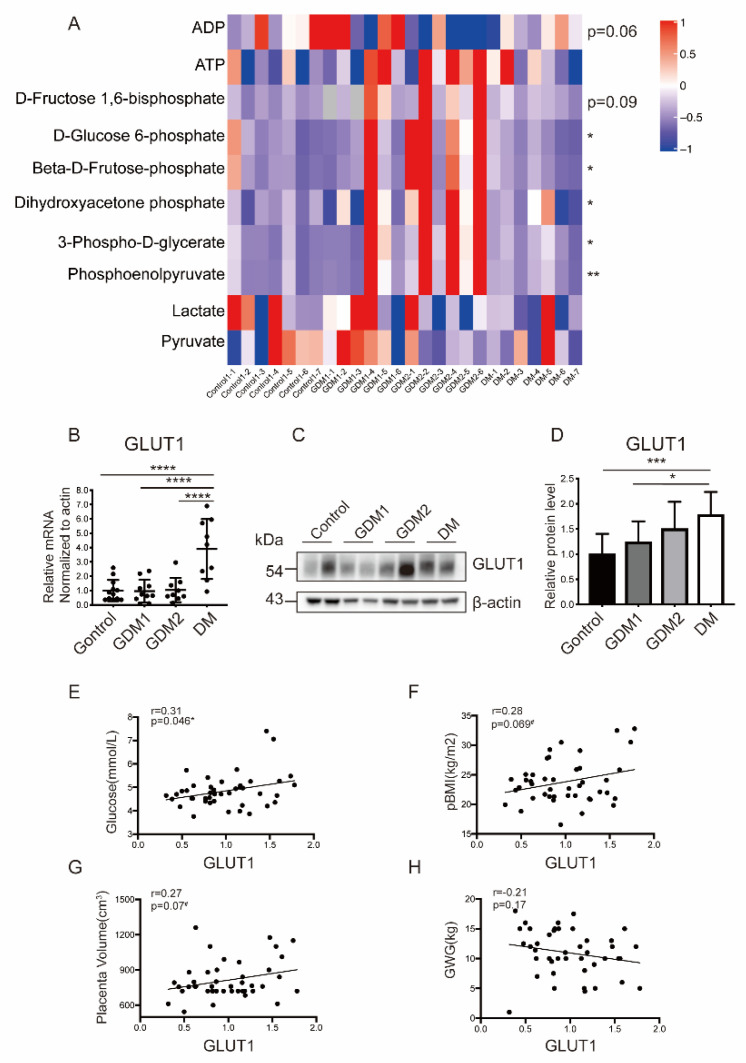
Placental glycolysis and GLUT1 expression were compromised under hyperglycemic conditions. (**A**) Heatmaps of the detected glycolytic intermediates among four groups. (**B**–**D**) Placental mRNA and protein levels of GLUT1. (**E**–**H**) Correlation analysis between GLUT1 and fasting glucose (**E**), p-BMI (**F**), placental volume (**G**), and GWG (**H**). Actin served as the internal controls. Data are the mean ± SEM. * *p* < 0.05, ** *p* < 0.01, *** *p* < 0.001, and **** *p* < 0.0001 by a one-way ANOVA test, followed by a post hoc test. p-BMI: Pre-pregnancy Body mass index and GWG: Gestational Weight Gain.

The overall glycolytic status was evaluated by examining key intermediates of glycolysis through targeted metabolomics. It was shown that the upstream mediates such as D-Glucose 6-phosphate, Beta-D-Fructose 6-phosphate, and D-Fructose 1,6-bisphosphate were significantly increased in HIP, especially in the GDM2 group (Figure 2A, Supplementary Figure S2A–F). The other candidates were consistent with this and followed by GDM1 and PGDM pregnancies. Furthermore, the GDM pregnancies were unique in their ability to generate ATP from high rates of glycolysis, and thus, more ATP production than the control group was found. 

Since the glycolytic intermediates showed obvious changes among the four groups, it was necessary to further examine the expression profile of GLUT1, one of the most important glucose transporters for evaluating the glucose uptake efficiency. It was observed that GLUT1 was expressed most in PGDM (Figure 2B–D). A correlation analysis was conducted between GLUT1 and clinical indicators with significant differences (p-BMI, GWG, fasting glucose, and placenta volume). It was shown that the GLUT1 protein level was positively related with third trimester fasting glucose (r = 0.31, *p* = 0.045), p-BMI (r = 0.28, *p* = 0.069), and the placenta volume (r = 0.27, *p* = 0.07) but negatively related with GWG (r = −0.21, *p* = 0.17) (Figure 2E–H).

### 2.4. Hyperglycemia Inhibited AMPKα Activation and Induced p38MAPK Phosphorylation in Both Placental Tissues and In Vitro Trophoblasts

**Figure 3:**
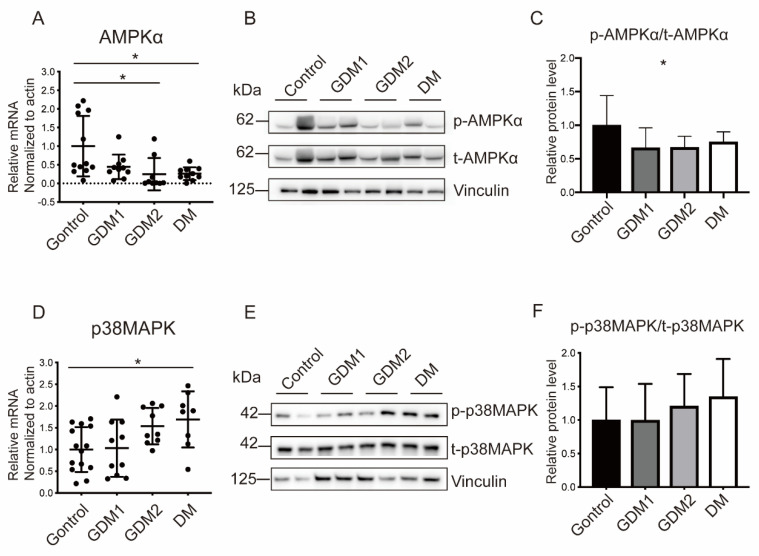
AMP-activated protein kinase signaling was activated accompanied by p38MAPK downregulation in placenta exposed to hyperglycemia. (**A**–**C**) Placental mRNA and protein levels of AMPKα. (**D**–**F**) Placental mRNA and protein levels of p38MAPK. Actin and Vinculin served as the internal controls. Data are the mean ± SEM. * *p* < 0.05 by a one-way ANOVA test, followed by a post hoc test.

AMPKα has emerged as a master regulator of cellular energy metabolism and can be activated by cellular stress, including glucose deprivation. To investigate the involvement of AMPKα in the HIP, RT-PCR and WB were performed in placenta tissues and found that it was obviously inhibited in GDM2 or PGDM pregnancies (Figure 3A–C). However, the stress-activated protein p38MAPK was highly phosphorylated, responding to OS (Figure 3D–F).

**Figure 4:**
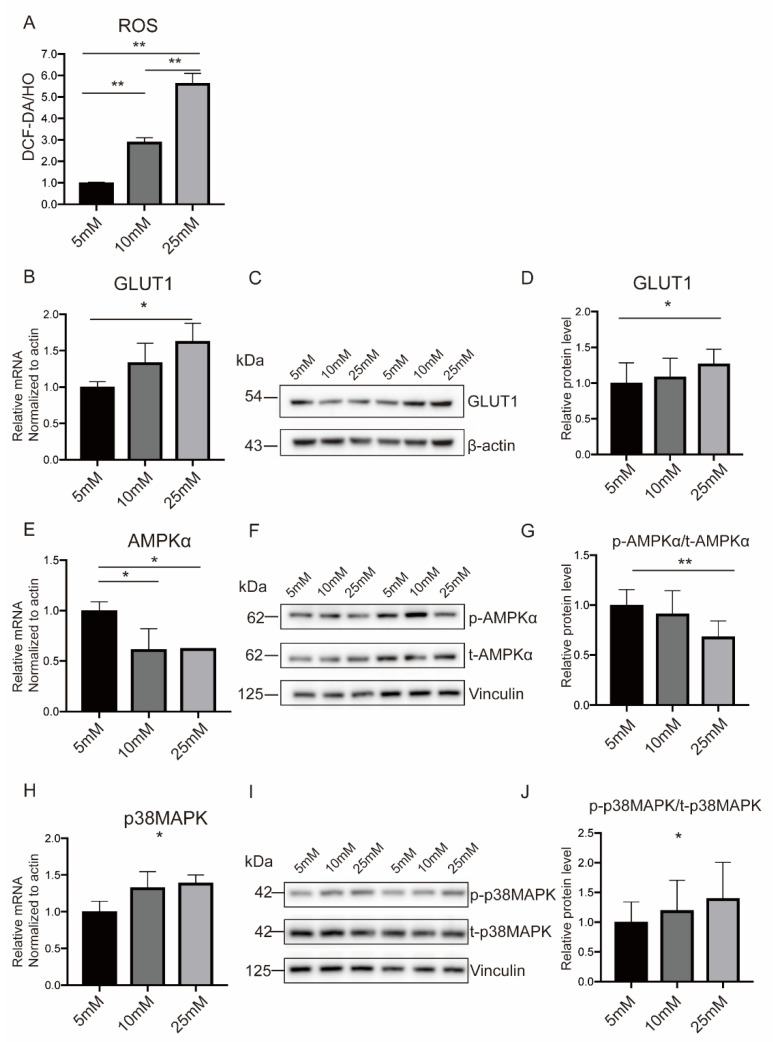
The oxidative status and expression levels of AMPKα, p38MAPK, and GLUT1 were altered in in vitro trophoblasts cultured in a high-glucose medium. (**A**) Intracellular ROS level in normal or high-glucose medium. (**B**–**J**) Cellular mRNA and protein levels of AMPKα (**B**–**D**), p38MAPK (**E**–**G**), and GLUT1 (**H**–**J**) cultured in normal or high-glucose medium. Actin and Vinculin served as the internal controls. Data are the mean ± SEM. * *p* < 0.05 and ** *p* < 0.01 by a one-way ANOVA test, followed by a post hoc test.

BeWo cells were fused spontaneously to form syncytiotrophoblast (STB) induced by Forskolin (FSK), as indicated by an increased expression of Syncytin2 and h-CGβ (Supplementary Figure S3A–D). Then, the cells were stimulated with a high-glucose medium to induce metabolic activity. High glucose (10 mM and 25 mM) tends to induce more ROS formation and improve OS responses (Figure 4A). In addition, the decreased antiapoptotic molecule (BCL2L2) and increased proapoptotic molecules (BAX, BAD, and BAK) in high-glucose surroundings, especially under 25 mM, simulated for PGDM pregnancies further demonstrated the augmented OS level (Supplementary Figure S4A–D). Consistent with the above results, AMPKα was downregulated, while p38MAPK and GLUT1 showed opposite trends with high-glucose stimulation (Figure 4B–J). 

### 2.5. p38MAPK Mediated Hyperglycemia-Stimulated GLUT1 Expression and Apoptosis in BeWo Cells 

**Figure 5:**
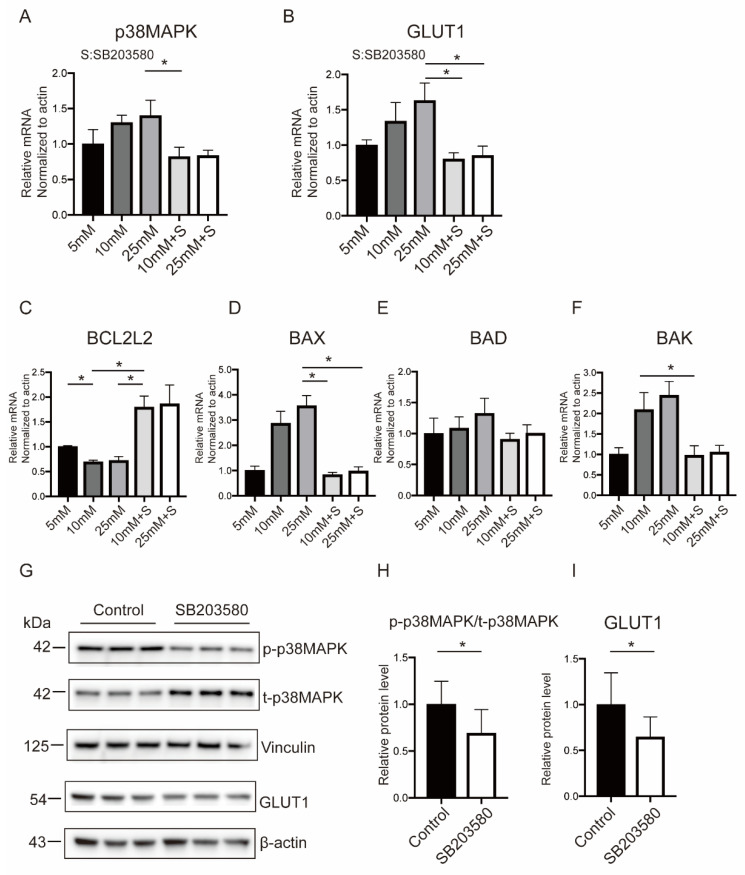
p38MAPK inhibition is associated with decreased GLUT1 and alleviated OS responses. (**A**–**F**) Cellular mRNA levels of p38MAPK (**A**), GLUT1 (B), and apoptotic molecules (**C**–**F**) in BeWo cells handled with the p38MAPK antagonist. (**G**–**I**) Cellular protein levels of p38MAPK and GLUT1 by adding the p38MAPK antagonist. Actin and Vinculin served as the internal controls. Data are the mean ± SEM. * *p* < 0.05 by a one-way ANOVA test, followed by a post hoc test. S: SB203580.

The roles of p38MAPK on GLUT1 expression and OS-mediated apoptosis were investigated using siRNA and SB203580, an inhibitor of p38 phosphorylation. It was found that the interventions could diminish the phosphorylation of p38MAPK effectively in BeWo cells exposed to a high-glucose medium (Figure 5A and Supplementary Figure S5A). Specifically, it could significantly suppress GLUT1 expression, making it equal to or even lower than the normal glucose level (Figure 5B and Supplementary Figure S5B). In addition, the mRNA level of BCL2L2 was restored, and simultaneously, the pro-apoptosis proteins (BAX, BAD, and BAK) were further decreased after p38MAPK inhibition (Figure 5C–F and Supplementary Figure S5C–F). The Western blot results were consistent with the previous findings when BeWo cells were cultured in the normal glucose medium with and without siRNA or SB203580 (Figure 5G–I and Supplementary Figure S5G–I). Together, these results indicated that p38MAPK was involved in the regulation of GLUT1 and OS responses.

### 2.6. Hyperglycemia-Related OS Augment Activated p38MAPK Pathway through AMPKα

**Figure 6:**
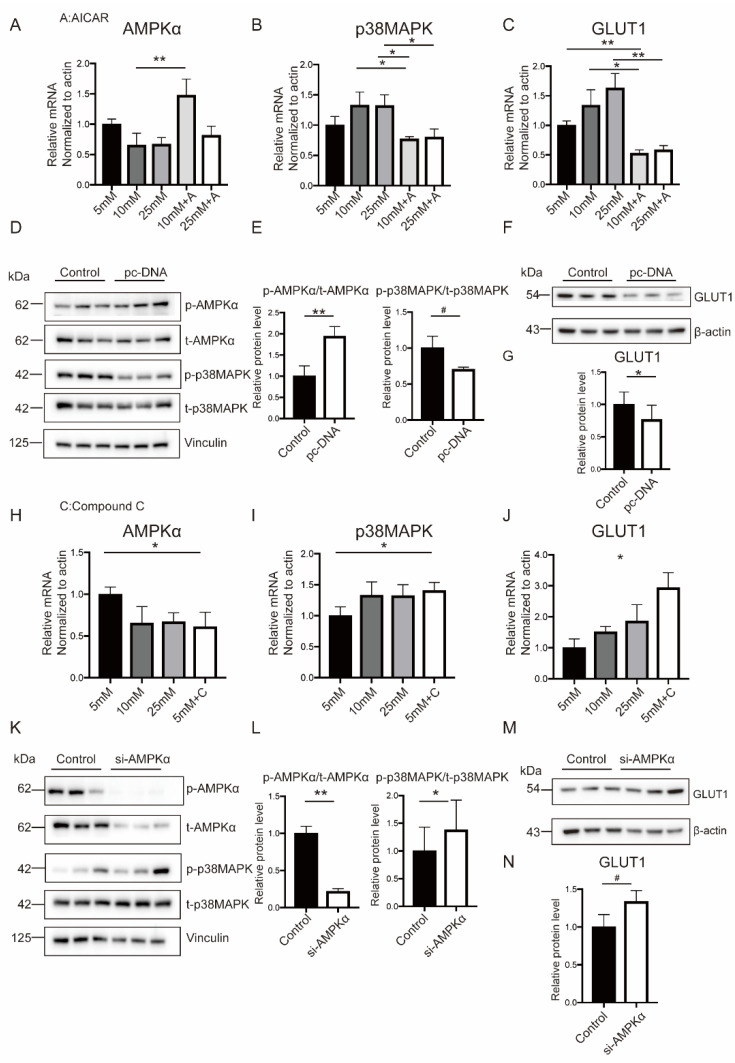
p38MAPK modulates glucose uptake and OS responses in an AMPKα-dependent manner. (**A**–**C**) Cellular mRNA levels of AMPKα (**A**), p38MAPK (**B**), and GLUT1 (**C**)) in BeWo cells treated with the AMPKα agonist. (**D**–**G**) Cellular protein levels of AMPKα, p38MAPK, and GLUT1 in BeWo cells transfected with negative or AMPKα-DN plasmid. (**H**–**J**) Cellular mRNA levels of AMPKα (**H**), p38MAPK (**I**), and GLUT1 (**J**)) in BeWo cells treated with the AMPKα inhibitor. (**K**–**N**) Cellular protein levels of AMPKα, p38MAPK, and GLUT1 in BeWo cells transfected with siRNA against AMPKα or scramble siRNA. Actin and Vinculin served as the internal controls. Data are the mean ± SEM. # *p* < 0.1, * *p* < 0.05, and ** *p* < 0.01 by a one-way ANOVA test, followed by a post hoc test. A: AICAR. C: Compound C.

Growing evidence showed that AMPKα was involved in glucose uptake through p38MAPK, particularly in tumor cells. Given the similarities between tumor and placental tissue in energy metabolism and proliferation, it was rational to hypothesize that p38MAPK acted as a downstream regulator in GLUT1 expression. As shown in Figure 6A–C, treating BeWo with AICAR, an agonist of AMPKα, could effectively inhibit p38MAPK phosphorylation and GLUT1 expression, followed by attenuated proapoptotic effects reflected in decreased mRNA levels of BAX, BAD, and BAK (Supplementary Figure S6F–I). The transfection of BeWo cells with negative or AMPKα-DN plasmid obtained the same results (Figure 6D–G). 

BeWo cells were cultured with Compound C to block AMPKα for establishing its casual role in regulating p38MAPK-mediated glucose uptake. As shown in Figure 6H–J, AMPKα inhibition significantly promoted p38MAPK and GLUT1 expression. This was also accompanied by the increased mRNA levels of BAX and BAD, which were even more than in normal conditions (Supplementary Figure S7E–H). AMPKα knockdown applying siRNA technology exhibited similar effects (Figure 6K–N and Supplementary Figure S7I–O). Together, these results indicated that p38MAPK functioned downstream of AMPKα in high-glucose-induced OS.

The regulatory mechanism was further verified at the tissue level, in which the placental explants were cultured in high-glucose DMEM medium and modified by AICAR, Compound C, and SB203580, respectively. The OS response was attenuated in the AICAR or SB203580 administration group, as directly indicated by a decreased ROS level, but without significant differences. The activity of SOD and T-AOC capacity were significantly decreased with AMPKα inactivation (Compound C) and significantly increased with p38MAPK dephosphorylation (SB203580), which were consistent with the ROS level (Supplementary Figure S8A–D). 

### 2.7. Trp53, Mknk2, Myc, and HIF1-α Targeted on p38MAPK Involving in GLUT1 Regulation

**Figure 7:**
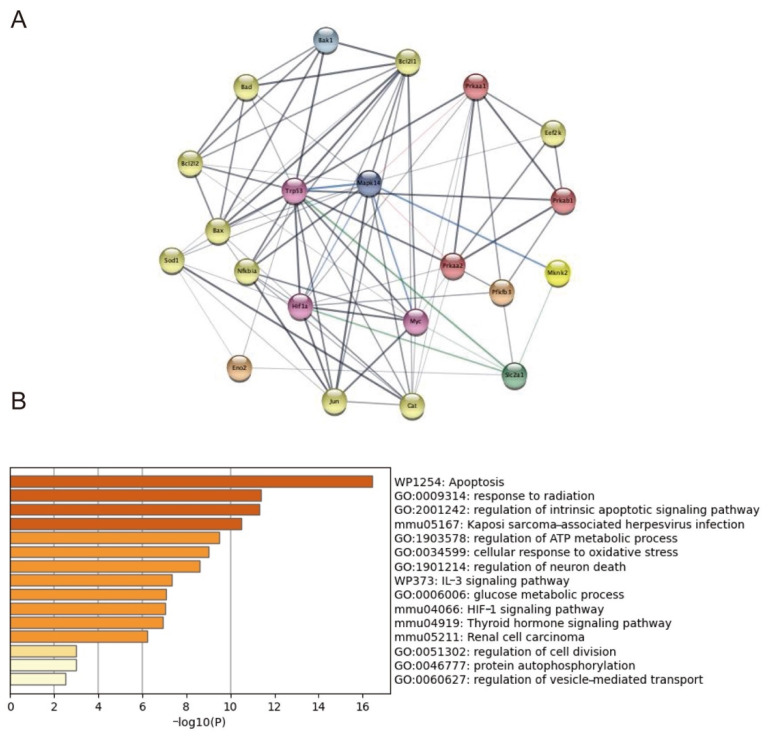
Potential molecules (Trp53, Mknk2, Myc, HIF1-α, Eno2, and Pfkfb3) targeted on p38MAPK in regulating AMPKα/p38MAPK/GLUT1 signaling cascades. The PPI (**A**) and functional enrichment analysis (**B**) of targeted genes generated from the GEO public dataset using IPA.

Chronic inflammation and insulin resistance are the main characteristics of diabetes and could be induced by LPS or TNFα in vitro or in animal models. Thus, transcriptome profiles generated from control and LPS-exposed mice placentae from the public dataset were downloaded. The original Ingenuity Pathway Analysis (IPA) found that AMPK phosphorylation was inhibited, and it activated the p38MAPK signaling pathway, acting as upstream regulators [31]. The protein–protein interaction network (PPI) analysis was performed through targeting genes involved in the OS signaling pathway. This indicates that Trp53, Mknk2, Myc, HIF1-α, Eno2, and Pfkfb3 may regulate p38MAPK phosphorylation and be involved in GLUT1 regulation. Then, the functional enrichment analysis was conducted, and the canonical pathways were mainly enriched on biological functions associated with apoptosis, oxidative stress, and metabolism (Figure 7).

## 3. Discussion

There was much data indicating that HIP-induced OS may entail nutrient transport disorders in the placenta [32] and biochemical disturbances in fetuses [33]. Consistent with previous research, this study found that the expression level and activity assays of antioxidants, mainly SOD1 and catalase, were significantly decreased in the HIP pregnancies most dominant in PGDM [34,35]. Additionally, OS-triggered apoptosis was verified in human and mouse placentae with a lower expression of BCL-2 [36]. Conversely, Joel Ram rez-Emilianos et al. found decreased oxidized substances in GDM placentae, suggesting a protective role against OS damage [37]. Despite strict dietary or insulin control, the glucose levels of HIP pregnancies were still much higher in the current study (4.71/5.17/5.19 vs. 4.66). The persistent and deleterious glucotoxicity may have already disrupted the adaptive mechanisms during the development of HIP.

Dysregulation in carbohydrate metabolism characterized by increased glycolysis/gluconeogenesis and decreased fatty acid metabolism were distinctive features of HIP [38]. These pregnancies showed higher glycolytic rates and increased GLUT1 expression. In line with this, glycolytic-related genes were upregulated in adipose tissue from women with GDM (PGK2 and GCK) [39]. Conversely, Amy M. Valent et.al. found that glycolytic activity was especially suppressed in primary cytotrophoblasts (CTB), and GLUT1 was downregulated in GDM [40]. Generally, discrepancies in the GLUT1 content were discovered in GDM populations, with the majority indicating an increased density [41,42,43,44]. The ambiguous results may be explained by the heterogeneity of participants, different diagnostic criteria, and glucose control levels. In PGDM, it was widely recognized that GLUT1 expression was elevated, mainly due to the enlarged surface area of nutrient exchange and efficient energy metabolism [13,14,15,16,45]. The placental volume was obviously larger in PGDM pregnancies and thus contributed to greater flows of glucose taken up. In the correlation analysis, GLUT1 was positively related to fasting glucose (*p* = 0.045) and placenta volume (*p* = 0.07). Combined with the unbalanced OS system and significantly decreased AMPKα phosphorylation, it is rational to detect an increased glycolytic rate and GLUT1 level under hyperglycemic conditions. It is worth noting that women with more advanced PGDM were likely to have severe underlying vascular diseases and abnormal placenta morphology, which may result in limited vascular resistance and nutrient availability [46,47]. Further experiments designed to differentiate the metabolic status of STB and CTB, respectively, were also needed. 

On the contrary, no correlation was found between GLUT1 and FBW [37]. In general, insulin therapy exerted beneficial effects on lowering macrosomia incidences (GDM2 and PGDM group) [48], and there were also no significant differences of FBW among four groups. Additionally, lipids may possibly serve as another strong contributor in modulating intrauterine fetal growth, with the presence of positive correlations between FBW and fatty acid transporter 6 (FATP6) [13].

Whether diabetes develops in the first or a later trimester, the placenta and fetus suffer from hyperglycemic stress and redundant ROS as a result of spiral artery remodeling with increased oxygen, and then, angiogenesis is promoted in the placenta to meet the fetal demands [16]. The accumulated cellular ATP and ROS in the HIP may inactivate AMPKα and simultaneously stimulate p38MAPK phosphorylation and apoptosis. The inverse correlation was also shown in p38α-targeted deletion mice and in vitro BeWo cells cultured in 5 mM and 25 mM medium [27,49]. In addition, adiponectin suppressed p38MAPK but activated AMPKα in high-glucose-induced apoptosis in NRK-52E cells [26]. The STB component is the main epithelium of human placenta responsible for nutrient transport, and thus, FSK was adopted to induce BeWo cell syncytialization to mimic the biological functions of STB in vivo. Similar results were obtained in cells with those in placental tissue after high-glucose management. Treating BeWo with p38MAPK inhibitors (SB203580) or adopting siRNA technologies could significantly suppress GLUT1 expression and relieve apoptosis. Furthermore, supplementing AMPKα antagonists (Compound C) or siRNA agents, the p38MAPK and GLUT1 were overexpressed and accompanied by enhanced apoptotic responses. The same conclusions were obtained by reverse validations with the addition of the AMPKα agonist (AICAR) and transfecting plasmids. Based on these results of the phenotype and mRNA levels, the cells only dealt with a normal medium to further validate the above mechanisms at the protein level and obtained the same conclusion. In addition, the placental explants were collected and detected the OS responses (ROS level, SOD and catalase activity, and T-AOC capacity) after modification of the AMPKα-p38MAPK cascades, which further confirmed the findings in the cell experiments. 

AMPKα can be inhibited through indirect implications on the AMP/ATP ratio or direct functions on SIRT1 or PP2A, leading to IR [50]. The AMPKα activators exhibited tremendous benefits in maintaining glucose metabolism homeostasis in diabetes and associated complications. Metformin is a widely used drug for T2D. It could increase the AMP/ATP ratio through inhibiting mitochondrial ATP synthesis, resulting in the activation of AMPKα, a reduction in hepatic glucose production and augmentation of insulin sensitivity [51]. Therefore, it is speculated that AMPKα activity improvement can provide promising results in HIP. Other metabolic effects medicated by AMPKα activators (such as Berberine, A-769662, and polyphenols) involve decreasing the body weight by acting on the satiety center or improved metabolic status [50]. This may help to prevent excessive GWG during gestation and avoid other pregnancy complications. Combined with its regulatory role on GLUT1 expression, more efficacious and safer agents, such as monoclonal antibodies to activate the AMPKα pathway in HIP, are warranted. The AMPKα agonist polyphenols also exerted antioxidant effects and a decreased incidence of cardiovascular disorder through generating NO in the PI3k/PKB pathway [52]. Suppressing NO synthesis could aggravate diabetes and complications by stimulating TGFα. This also provides more evidence for antioxidant therapy in HIP. Improved animal experiments or clinical trials are needed to mimic the translational gap.

Noteworthily, AMPKα and p38MAPK also show consistent changes in tumors and other tissues [28]. The differences may be associated with tissue-specific fashions, comprehensive regulatory networks, and heterogeneous microenvironments [16]. It is speculated that other pathways may be involved, such as mTOR [53]. In this study, although it was confirmed that p38MAPK was a downstream target of AMPKα in regulating GLUT1 expression, it could not conclude that the regulation was taking place by direct phosphorylation. The downloaded transcriptome profiles, which were based on the premise that AMPK phosphorylation was inhibited with the activated p38MAPK signaling pathway due to intrauterine inflammation [31], did indicate some new transcription factors involved in the regulation targeting on p38MAPK. Particularly, HIF-1α and its effector Pfkfb3 were activated in islets from individuals with T1DM and streptozotocin-induced diabetes mouse liver [54,55]. As a positive regulator of glycolysis, Pfkfb3 was suppressed by metformin, resulting in TLR4/NF-κB signaling inhibition and corrected OS responses [56]. Human umbilical cord-derived MSCs could reverse the high-glucose-stimulated ERK/MAPK signaling pathway, mainly targeting P53, Myc, and Mknk2 [57]. In addition, the antioxidant genes (SOD, catalase, and GSH-Px) were decreased, while biomarkers of glucose glycolysis (Gck and Eno2) were overexpressed in high-fat-fed rats. This could be reversed by instant dark tea intervention [58]. Summarily, the new transcription factors played potential roles in the regulation of OS induced by hyperglycemia and provided evidence for further basic research. 

GLUT3 may play an important role to regulate placental function combined with GLUT1. It was regulated by AMPKα and downregulated in pregnancies complicated by GDM, resulting in enhanced apoptosis in HTR8/SVneo cells [18]. GLUT3 was also regulated by p38MAPK in LP9M80-H-treated mice and thus correlated with the insulin signaling pathway [59]. In a high-fat diet (HFD)-induced rat or high-glucose-induced medium, the GLUT3 expression was increased and associated with the activation of hippocampal endoplasmic reticulum stress (ERS) and ERS-mediated apoptosis (Bax and Bcl2). The excessive ERS attenuated p38/ERK-CREB signaling pathways and activated NLRP3-IL-1β pathways [60,61]. In Sertoli cells with glucose deprivation, an increase in GLUT1 and decrease in GLUT3 expression were shown, accompanied by an activation of the AMPK, PI3K/PKB, and p38MAPK pathways. However, a possible participation of the AMPK- and p38MAPK-dependent pathways in the regulation of glucose uptake and GLUT1, but not GLUT3 expression, was found by using specific inhibitors [62]. Taken together, GLUT3 played an important role in glycolysis, ROS, or apoptosis regulation and was closely associated with the AMPK or p38MAPK pathway. Further research is needed to confirm its role through AMPKα-p38MAPK-GLUT1 cascades in regulating the metabolism and oxidative stress in HIP placentae.

**Figure 8:**
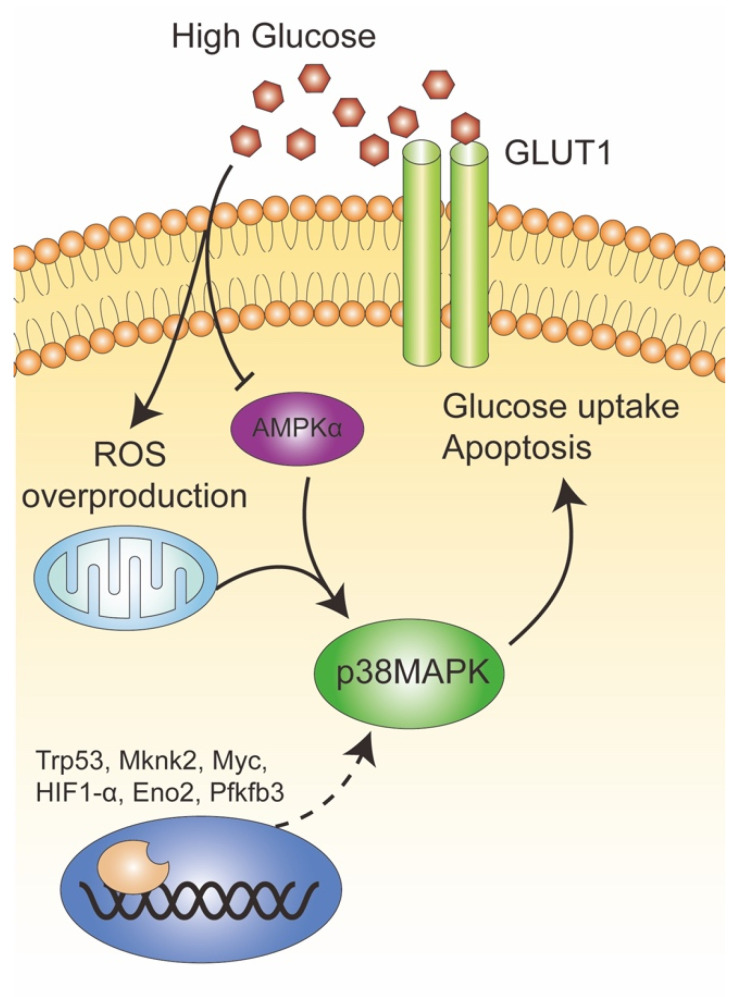
Schematic diagram: The mechanism of AMPKPα-p38MAPK signaling on GLUT1 regulation in trophoblast cells. AMPKα was inactivated, while p38MAPK and GLUT1 were upregulated in high-glucose-induced OS impairment accompanied by enhanced apoptosis. Potential molecules (Trp53, Mknk2, Myc, HIF1-α, Eno2, and Pfkfb3) may target p38MAPK and be involved in GLUT1 regulation. Solid arrow: established regulatory mechanism and dotted arrow: predicted regulation.

In the current study, pregnancies with different degrees of glucose impairment (GDM and PGDM) were enrolled, and GDM populations were further sub-grouped according to insulin treatments or not. In the context of clear medical interventions and glucose control levels, this study provided robust evidence for OS response and GLUT1 expression characteristics. Based on this, one novel pathway regulating GLUT1 expression through p38MAPK premised on AMPKα was demonstrated (Figure 8). This provided more evidence that AMPKα is a potential target for HIP. There were several limitations. Firstly, GLUT1 was differently expressed in the two plasma membranes of STB: microvillous membrane (MVM) and basal membrane (BM), and BM GLUT1 expression was positively correlated with FBW [63]. Therefore, further experiments designed to isolate the STB membrane and differentiate the metabolic status were needed. Secondly, it is necessary to explore deeper regulatory mechanisms based on newly discovered transcription factors. In addition, animal experiments were needed to verify the potential therapeutic effects of antioxidant agents or AMPKα activators on the maternal and fetal outcomes in the following study. 

## 4. Materials and Methods

### 4.1. Study Participants and Sample Collection

Placenta samples from women with pre-gestational diabetes mellitus (*n* = 10), gestational diabetes mellitus (*n* = 19), and normal pregnancies (*n* = 14) were collected at Peking University First Hospital between July 2019 and June 2021. All participants enrolled in this study had no history of preeclampsia, hypertension disorders, chronic diseases, smoking or drinking habits, fetal anomalies, intrauterine fetal growth restriction, and infections. The placenta samples were obtained within 30 min after cesarean sections, and fragments of villous were isolated from the basal plate at sites located 5 cm from the umbilical cord insertion site. The tissues were stored at −80 °C until further analysis.

This project was approved by the Ethics Committee of Peking University First Hospital (V2.0/201504.20), and informed consent was obtained from all participants.

### 4.2. Placental Explant Culture

Placental tissues were collected from uncomplicated pregnancies by cesarean sections and washed thoroughly in sterile PBS. After dissected into small pieces of approximately 0.5 mm^3^, the placental explants were cultured in DMEM (Gibco, Grand Island, NY, USA) supplemented with 10% fetal bovine serum (FBS, Gibco, MA, USA), 100 U/mL penicillin (Lonza, Basel, Switzerland), 100 U/mL streptomycin (Lonza), and 250 ng/mL amphotericin B (Lonza) at 37 °C. After being allowed to attach for 6–12 h, the explants were treated with 20 μM Compound C, 1 mM 5-Aminoimidazole-4-carboxamide ribonucleoside (AICAR), and 60 μM SB203580 for 8 h, 2 h, and 6 h, respectively. The placental explants were collected for the subsequent analysis after culturing for 36 h. 

### 4.3. Determination of Oxidative Stress Markers

Equal amounts of placental tissues were dissolved in RIPA buffer, and 10% of the total homogenate was used to detect OS-associated markers according to the manufacturer’s standard. Kits for the malondialdehyde content (MDA, A003), total antioxidant capacity (T-AOC, A015), activity assays of superoxide dismutase (SOD, A001), catalase (CAT, A007), and L-Glutathione (GSH, A006) were from Nanjing Jiancheng Bioengineering Institute (Nanjing, China). 

### 4.4. Measurement of Glycolytic Metabolites by LC-MS/MS 

The protein was collected by homogenate lysis. Briefly, a 100-mg sample was mixed with 1 mL cold methanol/acetonitrile/H2O (2:2:1, *v*/*v*/*v*) and sonicated at a low temperature (30 min/once, twice). After centrifugation, the supernatant was dried in a vacuum centrifuge and then redissolved in 100 μL acetonitrile/water (1:1, *v*/*v*) for LC-MS analysis. Analyses were performed using a UHPLC (1290 Infinity LC, Agilent Technologies, Palo Alto, CA, USA) coupled to a QTRAP (AB Sciex 5500) using an ACQUITY UPLC BEH Amide column (2.1 × 100 mm, 1.7 μm, Waters MS Technologies, Manchester, UK). The MS/MS Analysis (MRM) was performed in ESI-negative mode. Data acquisition and processing were accomplished using Multiquant software (AB SCIEX, Boston, MA, USA).

### 4.5. Cell Culture and Treatments

The human choriocarcinoma originated BeWo cell lines were purchased from the National Infrastructure of Cell Line Resource (NICR, Beijing, China) and maintained in Roswell Park Memorial Institute (RPMI) 1640 medium (Thermo Fisher Scientific, Grand Island, NY, USA) supplemented with 10% (*v*/*v*) FBS (Gibco, MA, USA), 100 U/mL penicillin (Lonza, Basel, Switzerland), 100 U/mL streptomycin (Lonza), and 250 ng/mL amphotericin B (Lonza) at 37 °C in a humidified atmosphere containing 5% CO_2_. The syncytialization of BeWo cells was induced by incubation with 20 μM FSK (Selleck, Houston, TX, USA) for 48 h and then incubated with 5 mM D-glucose (control group), 10 mM D-glucose (high-glucose group simulated for GDM), and 25 mM D-glucose (high-glucose group simulated for PGDM) for another 48 h. For the mechanism analysis, the AMPKα inhibitor Compound C and agonist AICAR were obtained from MedChemExpress (Monmouth Junction, NJ, USA), and the p38MAPK inhibitor SB203580 was from Selleck (Houston, TX, USA). After syncytialization induction, 20 μM Compound C, 1 mM AICAR, and 60 μM SB203580 were added to a fresh medium with different glucose concentrations for 8 h, 2 h, and 6 h separately. Subsequently, the chemical materials were removed and cells continued to be cultured in the fresh medium. 

### 4.6. Measurement of Intracellular ROS

Cells were seeded at 6000 cells/well in a 96-well plate. After attachment and syncytialization, the cells were incubated with 5 mM, 10 mM, and 25 mM glucose. The medium was updated every 24 h for a total of 96 h. Thereafter, all groups were treated with a mixture of DCF-DA (20 μM, Sigma-Aldrich, St. Louis, MO, USA) and Hoechst 33342 (HO, 2.5 μg/mL, Sigma-Aldrich, St. Louis, MO, USA) for 30 min to detect intracellular ROS and the corresponding number of viable cells. The fluorescence intensity was measured by a microplate reader after discarding the supernatant and PBS rinse. The excitation/emission wavelengths were 490/530 nm for DCF-DA and 340/425 nm for HO. The results were calculated as the ratio of DCF-DA/HO signals per well. All samples were performed in triplicate. The placental explants were cultured and digested into single-cell suspensions, and then, the ROS level was measured according to the manufacturer’s standard (E004, Nanjing Jiancheng Bioengineering Institute).

### 4.7. Transfection in BeWo Cells

The BeWo cells were cultured to 60–70% confluence and transiently transfected with a nonspecific negative control small interfering RNA (siRNA) or siRNA against genes encoding AMPKα and p38MAPK using Lipofectamine RNA iMax (Invitrogen, Karlsbad, CA, USA) in Opti-MEM reduced serum medium (Invitrogen, Karlsbad, CA, USA). After 24-h transfection, the BeWo cells were treated with fresh medium containing 5 mM, 10 mM, or 25 mM glucose for further analysis.

For AMPKα overexpress experiments, the BeWo cells were seeded to 70–80% confluence and transiently transfected with pc-DNA as the negative control and AMPKα-DN by Lipofectamine 3000 (Invitrogen, Karlsbad, CA, USA) according to the manufacturer’s protocol. The medium was replaced with a fresh medium after 4–6 h of transfection and treated with a normal or high-glucose medium after 24 h. Cells were harvested after 48 h for RT-PCR analysis and 72 h for Western blot analysis.

### 4.8. RNA Isolation and Reverse-Transcription Polymerase Chain Reaction (RT-PCR)

Total RNA from the cultured cells or tissue samples was extracted using TRIzol reagent (Invitrogen, Carlsbad, CA, USA) according to the manufacturer’s instructions, and cDNA was synthesized from 2 ug of RNA using the FastKing RT Kit with DNase (Tiangen Biotech, Beijing, China). The gene expression analysis was evaluated by RT-PCR using the ABI Power SYBR Green gene expression system (Applied Biosystems, Waltham, MA, USA) on an ABI 7500 sequence detection system. The primer sequences used are listed in Supplementary Table S1. The relative expression levels of mRNA were normalized to β-actin, and the fold changes were calculated using the 2^−(ΔΔCt)^ method.

### 4.9. Western Blot Analysis 

The tissue samples or cells were washed in PBS and lysed in cold RIPA buffer (KeyGen Biotech, Nanjing, China) supplemented with a protease inhibitor cocktail (Sigma Aldrich, Merck Millipore, Boston, MA, USA) and phosphatase inhibitors (Roche, Mannheim, Germany). The protein concentration was determined using a Pierce BCA Assay kit (Thermo Fisher Scientific, Inc.).

Immunoblotting was performed with primary antibodies against AMPKα (5831, Cell Signaling Technology, Beverly, MA, USA), AMPKα phosphorylated at Thr172 (2535, Cell Signaling Technology, Beverly, MA, USA), p38MAPK (8690, Cell Signaling Technology, Beverly, MA, USA), p38MAPK phosphorylated at Thr180/Tyr182 (4511, Cell Signaling Technology, Beverly, MA, USA), SOD1 (37385, Cell Signaling Technology, Beverly, MA, USA), catalase (12980, Cell Signaling Technology, Beverly, MA, USA), GLUT1 (AP21407B, Abcepta, Suzhou, Jiangsu, China), HERV-FRD (AP13018A, Abcepta, Jiangsu, China), Vinculin (ab129002, Abcam, Cambridge, UK), and β-actin (4970, Cell Signaling Technology, Beverly, MA, USA) overnight at 4 °C. Subsequently, the membranes were further incubated with horseradish peroxidase (HRP)-conjugated secondary antibody (7074, Cell Signaling Technology, Beverly, MA, USA). The signals were visualized using an ECL kit (Merck Millipore) and the Syngene GeneGenius gel imaging system (Syngene, Cambridge, UK). Independent experiments were repeated at least three times using cultured cells. Detected bands were analyzed with densitometry using ImageJ software.

### 4.10. Protein–Protein Interaction Network and Functional Enrichment Analysis

Gene expression data were collected from public datasets at the Gene Expression Omnibus database (GEO: GSE151728) (http://www.ncbi.nlm.nih.gov/geo/, accessed on 7 February 2022). After identifying target genes, the protein–protein interaction network (PPI) analysis and enrichment analysis were performed on the STRING database (http://string-db.org, accessed on 7 February 2022) and Metascape, respectively (http://metascape.org/gp/index.html#/main/step1, accessed on 7 February 2022).

### 4.11. Statistical Analysis

The results were expressed as the mean ± standard deviation (SD). Comparisons were performed by the Student’s *t*-test or the one-way ANOVA, followed by a post hoc test using SPSS version 26.0 (SPSS Inc., Chicago, IL, USA). The molecular experiments were conducted independently at least three times. For the association analysis between the GLUT1 expression level and clinical factors, Person’s correlation coefficient was adopted with the following selected parameters: maternal pre-pregnancy body mass index (p-BMI), gestational weight gain (GWG), fasting glucose in the third trimester, placental volume, fetal birth weight (FBW), and placental ratio and was considered statistically significant at a *p*-value of < 0.05. Statistically significant differences were shown as follows: **** *p* < 0.0001, *** *p* < 0.001, ** *p* < 0.01, * *p* < 0.05, and # *p* < 0.1.

## 5. Conclusions

In the HIP groups, the antioxidant substances were decreased concomitantly with overexpressed proapoptotic molecules. The GLUT1 expression was also higher and significantly correlated with the third trimester glucose level. It could be regulated by AMPKα-p38MAPK cascades and exert influences on placental transport functions. These findings supplemented new evidence for the potential therapeutic effect of AMPKα on alleviating diabetes progression in pregnancy.

## Figures and Tables

**Table 1:** Clinical characteristics of the pregnant women enrolled in this study.

	Control (*n* = 14)	GDM1 (*n* = 10)	GDM2 (*n* = 9)	PGDM (*n* = 10)	*p*-Value
Age (years)	32.78 ± 3.577	33.70 ± 4.448	35.67 ± 3.605	34.70 ± 4.877	0.4
Gestational age (Weeks)	38.64 ± 0.745	38.80 ± 0.632	38.89 ± 0.333	38.60 ± 0.516	0.681
p-BMI (kg/m^2^)	21.68 ± 1.765	22.27 ± 2.491	26.56 ± 3.092	25.77 ± 4.803	0.001 **
GWG (kg)	13.37 ± 4.239	11.40 ± 2.989	9.49 ± 2.875	8.45 ± 3.218	0.008 **
GLU0 (mmol/L) a	4.51 ± 0.180	5.17 ± 0.415	5.53 ± 0.420	-	<0.0001 ****
GLU1 (mmol/L) a	7.88 ± 1.161	10.23 ± 1.335	10.00 ± 1.321	-	<0.0001 ****
GLU2 (mmol/L) a	6.57 ± 0.873	9.21 ± 1.086	8.15 ± 1.520	-	<0.0001 ****
AUC	13.42 ± 1.495	17.42 ± 1.402	16.84 ± 1.952	-	<0.0001 ****
Third trimester glucose (mmol/L)	4.48 ± 0.371	4.71 ± 0.463	5.17 ± 0.925	5.19 ± 0.838	0.035 *
Fetal birth weight (g)	3439.28 ± 330.657	3580.00 ± 512.809	3582.78 ± 256.065	3350.00 ± 324.414	0.509
Height (cm)	50.21 ± 0.975	50.50 ± 1.581	50.33 ± 1.000	50.28 ± 1.054	0.858
Ponderal Index (kg/m^3^)	2.91 ± 0.143	2.73 ± 0.260	2.81 ± 0.133	2.66 ± 0.200	0.393
Head circumference (cm)	33.96 ± 0.499	34.07 ± 0.861	34.39 ± 0.928	34.11 ± 0.782	0.626
Placenta weight (g)	580.00 ± 80.288	641.33 ± 158.338	602.22 ± 69.061	630.00 ± 92.736	0.509
Placenta volume (cm^3^)	704.86 ± 71.471	778.00 ± 90.985	954.89 ± 198.515	870.30 ± 158.711	<0.0001 ****
Placental coefficient	0.169 ± 0.023	0.186 ± 0.068	0.169 ± 0.025	0.192 ± 0.030	0.456

Data was expressed as the mean ± SD, **** *p* < 0.0001, ** *p* < 0.01 and * *p* < 0.05. p-BMI: Pre-pregnancy Body Mass Index, GWG: Gestational Weight Gain, AUC: Area Under Curve, a: Results of the 75 g Oral Glucose Tolerance Test (OGTT), and GLU: Glucose.

## Data Availability

Not applicable.

## Supplementary Materials

The supporting information can be downloaded at https://www.mdpi.com/article/10.3390/ijms23158572/s1.
