# Supplementary material for: Hyperglycemia in Pregnancy-Associated Oxidative Stress Augments Altered Placental Glucose Transporter 1 Trafficking via AMPKα/p38MAPK Signaling Cascade
Source: Int J Mol Sci. 2022 Aug 2;23(15):8572. doi: 10.3390/ijms23158572 (PMC9369398; doi:10.3390/ijms23158572)
Supplement: Supplementary file 1 [file ijms-23-08572-s001.zip › ijms-1781381-supplementary.pdf]

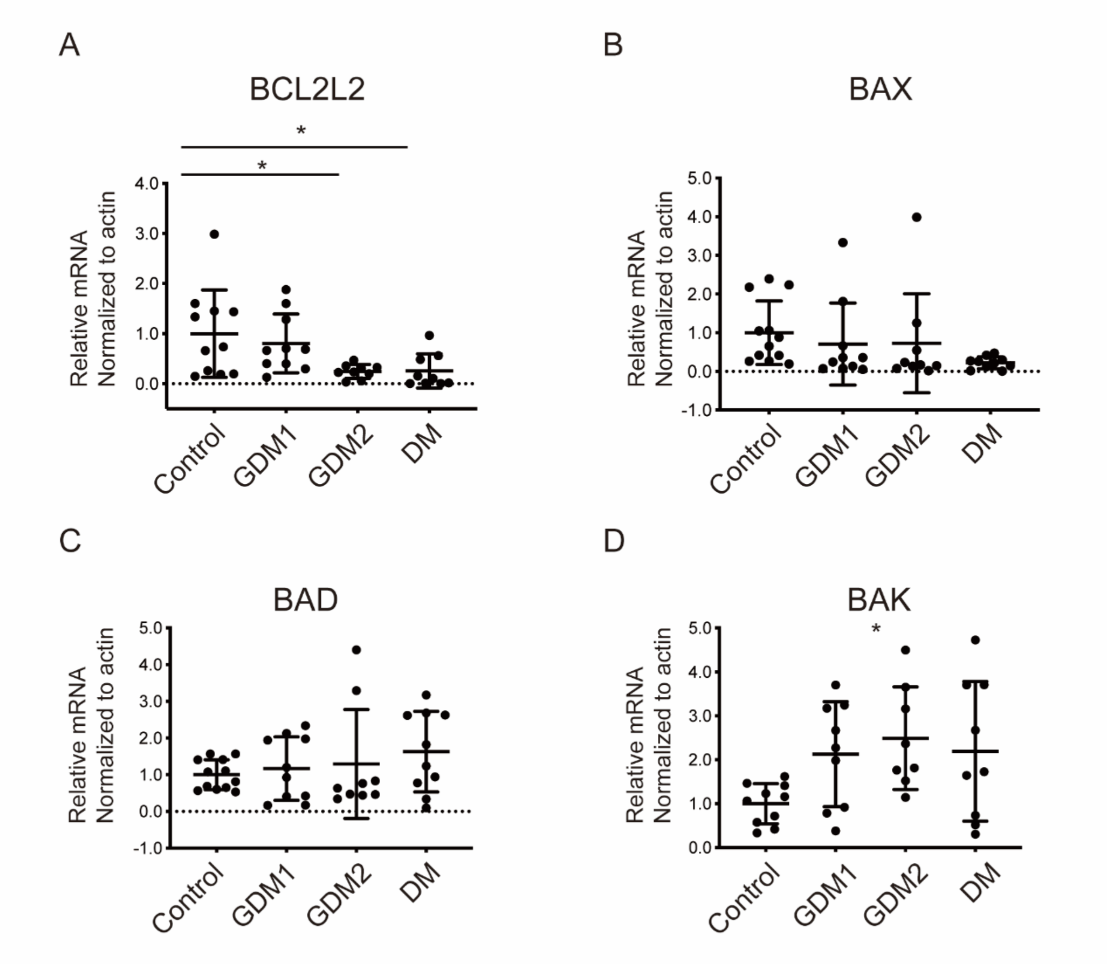

Figure S1: The pro-apoptotic status was enhanced in HIP pregnancies. (A-D) Placental mRNA levels of anti-apoptotic molecule BCL2L2 (A) and pro-apoptotic BAX (B), BAD (C) and BAK (D). Actin served as internal controls. Data are mean ± SEM. *P<0.05 by one-way ANOVA test followed by a post hoc test.

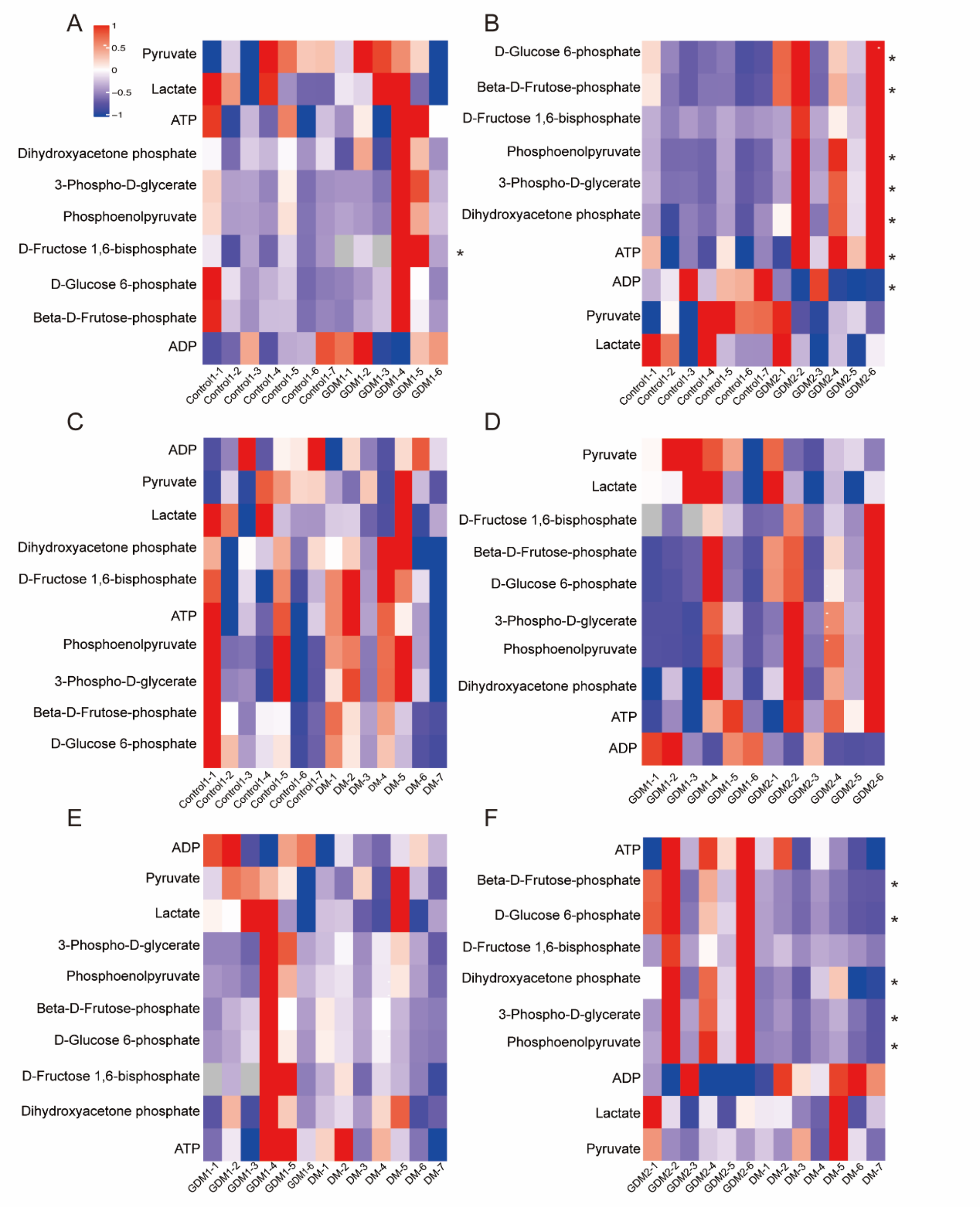

Figure S2: Placental glycolysis was upregulated under hyperglycemia conditions especially among GDM2 and PGDM pregnancies. (A-F) Heatmaps of the detected glycolytic intermediates of pairwise comparisons between control and GDM1 (A), control and GDM2 (B), control and PGDM (C), GDM1 and GDM2 (D), GDM1 and PGDM (E) and GDM2 and PGDM (F). *P < 0.05 defined by unpaired Student's t test.

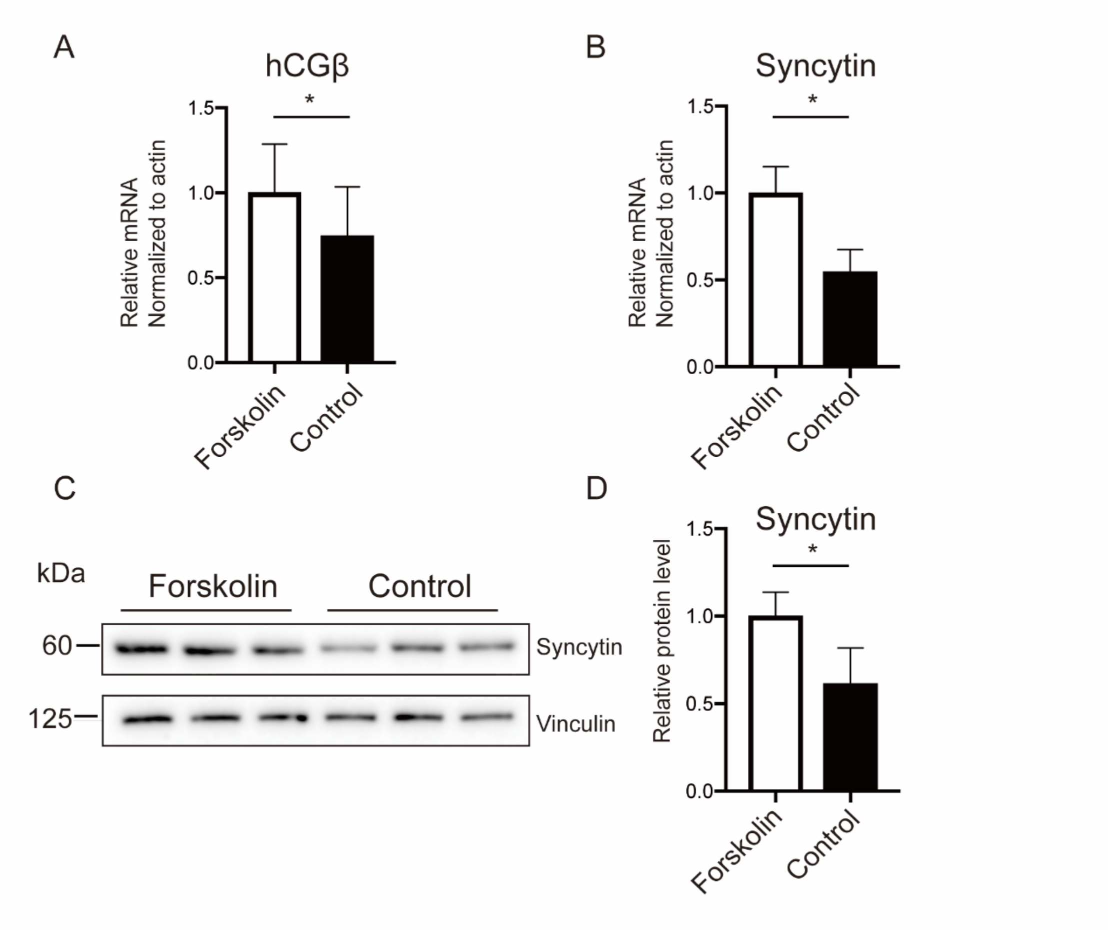

Figure S3: Bewo cells syncytialization was induced by Forskolin *in vitro*. (A-B) Intracellular mRNA levels of h-CGβ and Syncytin2 between control and FSK-induced syncutialization cells. (C-D) Intracellular protein level of Syncytin2 between control and FSK-induced syncutialization cells. Actin served as internal controls. Data are mean ± SEM. *P<0.05 by paired Student's t test.

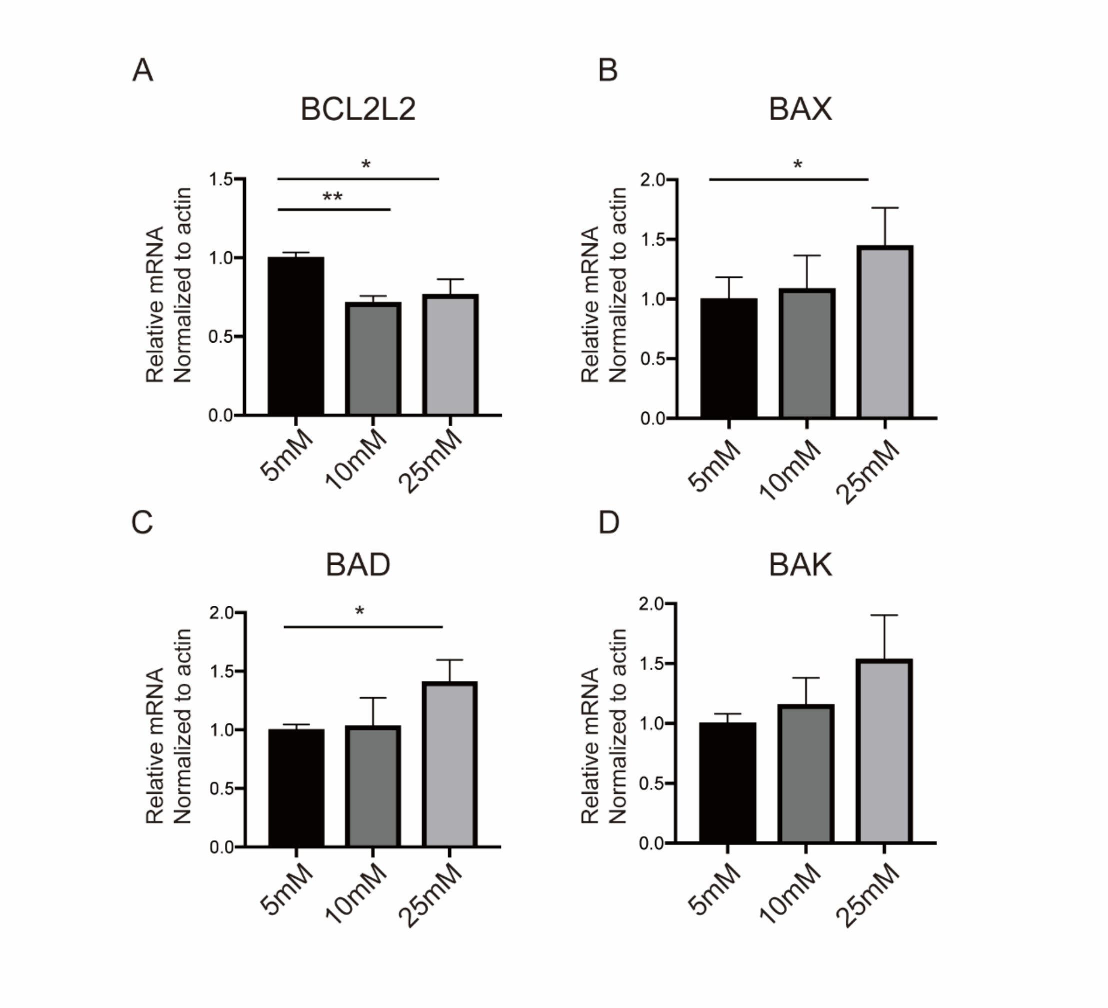

Figure S4: The pro-apoptotic status was augment in in vitro trophoblasts after high glucose stimulation. (A-D) Cellular mRNA levels of anti-apoptotic molecule BCL2L2 (A) and pro-apoptotic BAX (B), BAD (C) and BAK (D) in normal or high glucose medium. Actin served as internal controls. Data are mean ± SEM. *P<0.05 and **P<0.01 by one-way ANOVA test followed by a post hoc test.

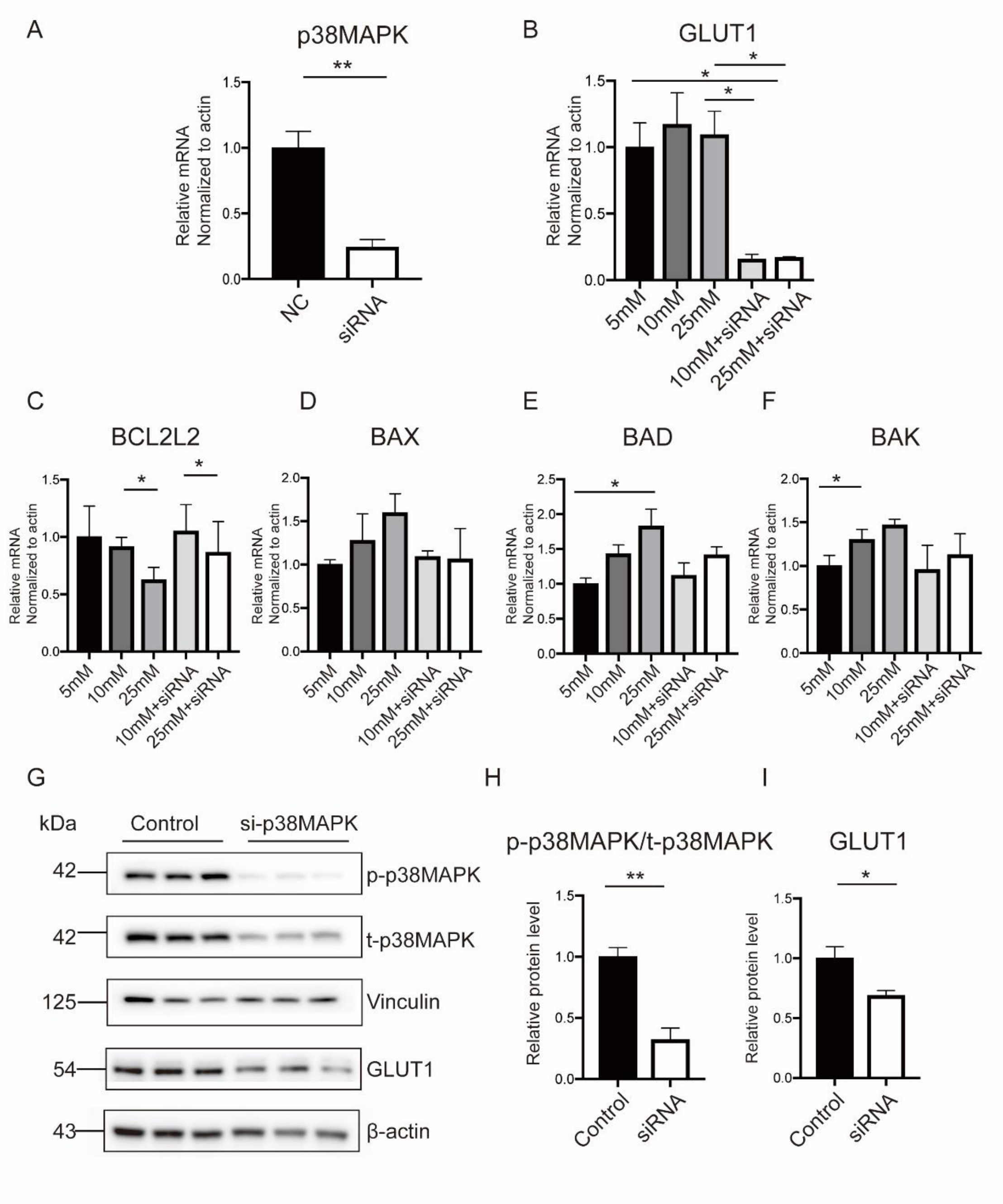

Figure S5: GLUT1 and pro-apoptotic molecules were downregulated after p38MAPK interfering. (A-F) Cellular mRNA levels of p38MAPK (A), GLUT1 (B) and apoptotic molecules (C-F) in BeWo cells transfected with siRNA agent against p38MAPK or scramble siRNA. (G-I) Cellular protein levels of p38MAPK and GLUT1 by siRNA interfering against p38MAPK or negative control. Actin and vinculin served as internal controls. Data are mean ± SEM. *P<0.05 and **P<0.01 by one-way ANOVA test followed by a post hoc test.

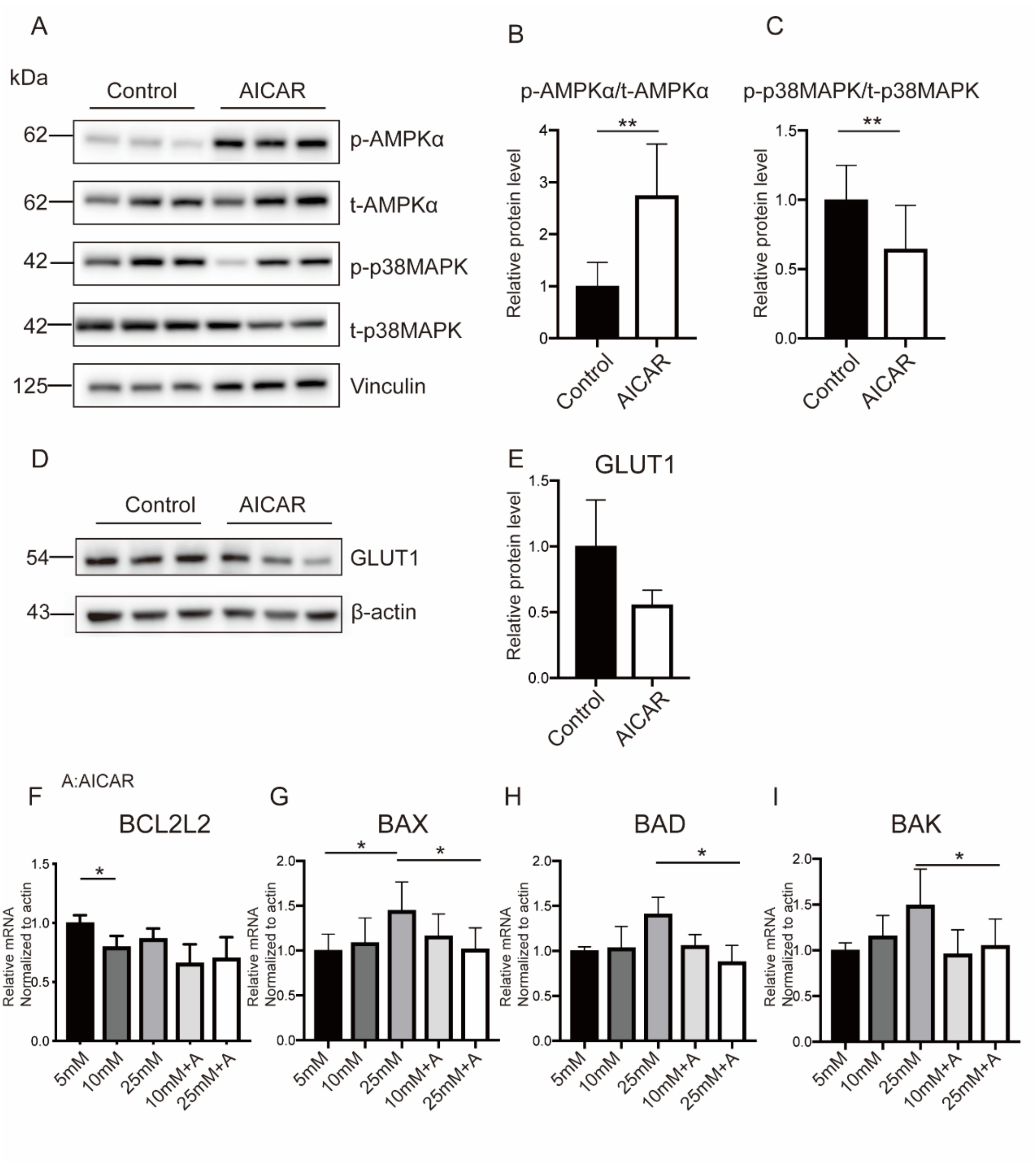

Figure S6: AMPKα overexpression decreases p38MAPK phosphorylation, GLUT1 level and apoptosis. (A-E) Cellular protein levels of AMPKα (A, B), p38MAPK (A,C) and GLUT1 (D,E) in BeWo cells treated with AMPKα agonist. (F-I) Cellular mRNA levels of apoptotic substances in BeWo cells treated with AMPKα agonist. Actin and vinculin served as internal controls. Data are mean ± SEM. *P<0.05 and **P<0.01 by one-way ANOVA test followed by a post hoc test. A: AICAR.

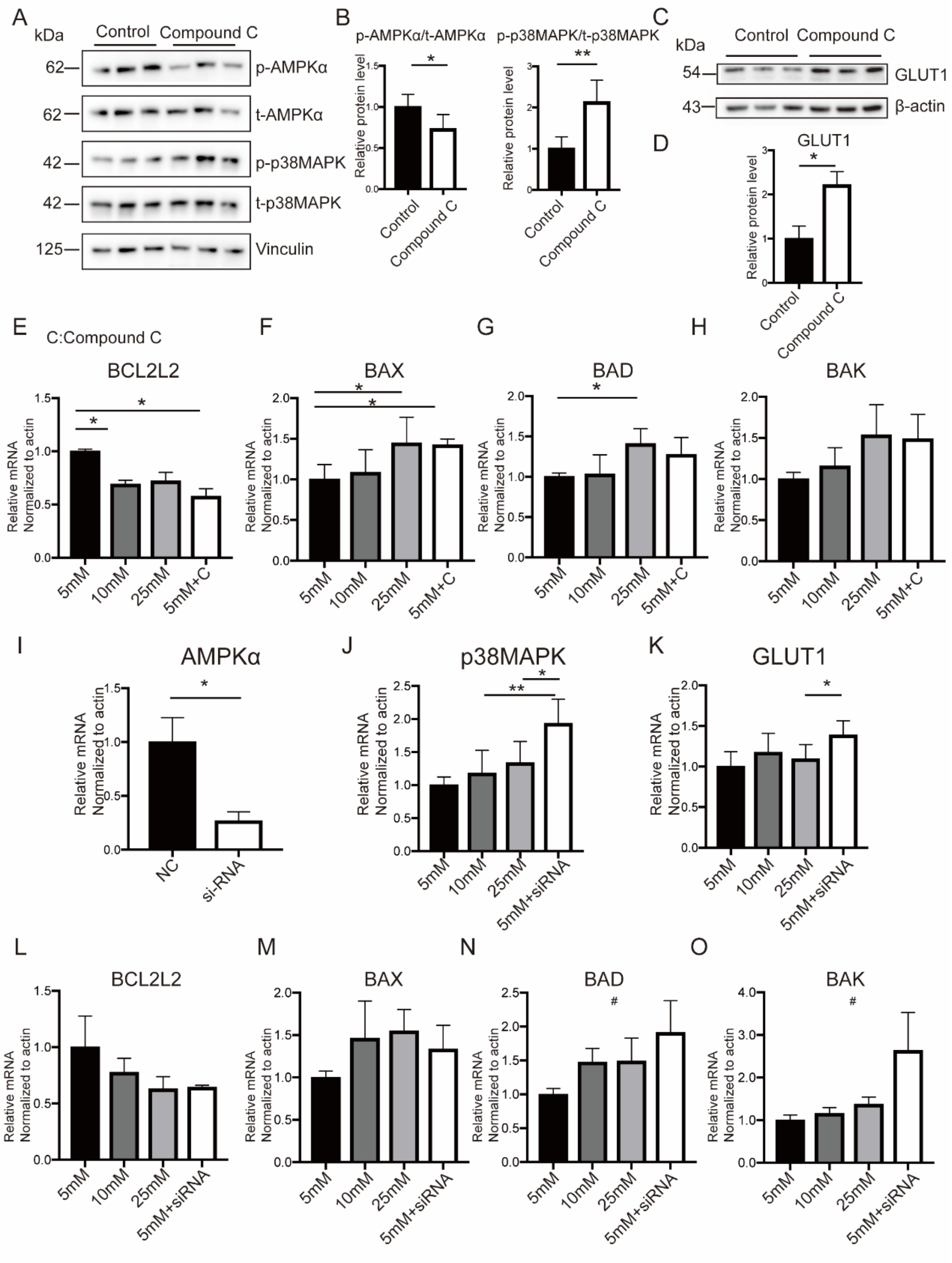

Figure S7: AMPKα inhibition could activate p38MAPK and GLUT1 expression and promote apoptosis. (A-D) Cellular protein levels of AMPKα, p38MAPK and GLUT1 in BeWo cells treated with AMPKα antagonist. (E-H) Cellular mRNA levels of apoptotic substances in BeWo cells treated with AMPKα antagonist. (I-O) Cellular mRNA levels of AMPKα (I), p38MAPK (J), GLUT1 (K), anti-apoptotic BCL2L2 (L) and pro-apoptotic molecules (M-O) in BeWo cells transfected with siRNA agents against AMPKα or scramble siRNA. Actin and vinculin served as internal controls. Data are mean ± SEM. *P<0.05 and **P<0.01 by one-way

ANOVA test followed by a post hoc test. C: Compound.

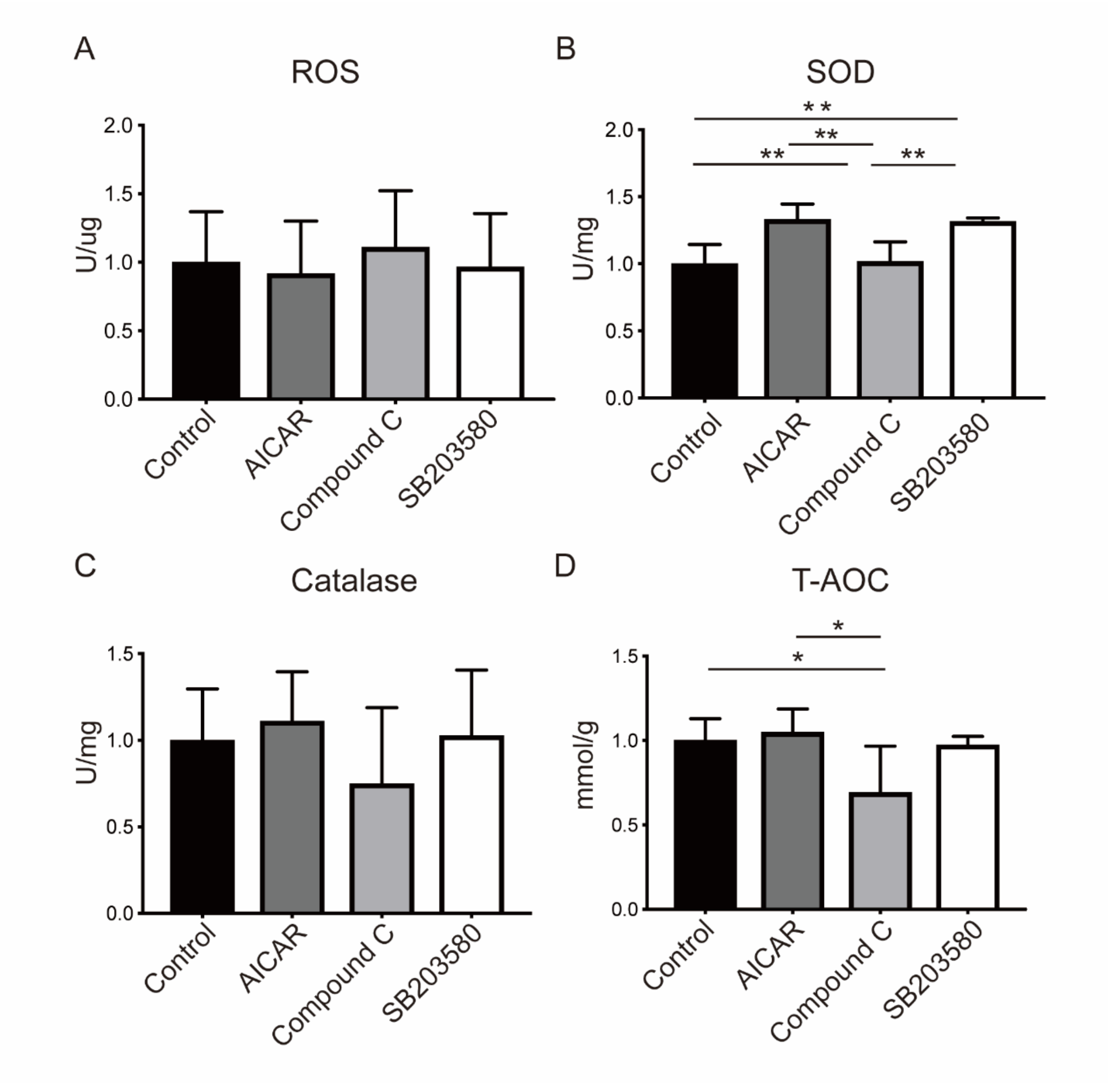

Figure S8: The OS responses were changed after modification of AMPKα-p38MAPK signaling in placental explants. (A) The level of ROS. (B-C) The activity of SOD and Catalase. (D) The capacity of antioxidant. Data are mean ± SEM. *P<0.05 and **P<0.01 by one-way ANOVA test followed by a post hoc test.

Gene	Species	Forward	Reverse
SOD1	Human	GGTGGGCCAAAGGATGAAGAG	CCACAAGCCAAACGACTTCC
GCLC	Human	GGAGGAAACCAAGCGCCAT	CTTGACGGCGTGGTAGATGT
GPX1	Human	CAGTCGGTGTATGCCTTCTCG	CAGTCGGTGTATGCCTTCTCG
BCL2L2	Human	TTTGGTTCGGCTTTATCAGG	GCAAACAGTGTGGCTTCAAA
BAX	Human	CATTCTACCTGAGGCCAGGA	CCCATCTCTTAGGGTGCTGA
BAD	Human	CCCAGAGTTTGAGCCGAGTG	CCCATCCCTTCGTCGTCCT
BAK	Human	GTTTTCCGCAGCTACGTTTTT	GCAGAGGTAAGGTGACCATCTC
AMPKα	Human	ACCAGGTGATCAGCACTCCA	TCTCTTCAACCCGTCCATGC
p38MAPK	Human	ATGCCGAAGATGAACTTTGC	TCTTATCTGAGTCCAATACAAGCATC
GLUT1	Human	ATTGGCTCCGGTATCGTCAAC	GCTCAGATAGGACATCCAGGGTA
β-actin	Human	CCTTGCCATCCTAAAAGCC	CACGAAAGCAATGCTATCAC

Supplementary Table S1: Primer sequences of RT-PCR used in this study.
